# Pharmacogenetics of Treatment Response in Psoriatic Arthritis

**DOI:** 10.1007/s11926-015-0518-z

**Published:** 2015-05-17

**Authors:** Meghna Jani, Anne Barton, Pauline Ho

**Affiliations:** Arthritis Research UK Centre for Genetics and Genomics; Centre for Musculoskeletal Research, University of Manchester, Room 2.704, Stopford Building, Oxford Road, Manchester, M13 9PT UK; National Institute of Health Research Manchester Musculoskeletal Biomedical Research Unit, Central Manchester Foundation Trust and University of Manchester, Manchester Academic Health Science, Manchester, UK; The Kellgren Centre for Rheumatology, Central Manchester Foundation Trust, Manchester Royal Infirmary, Oxford Road, Manchester, M13 9WL UK

**Keywords:** Psoriatic arthritis, Pharmacogenetics, Drug response, Polymorphisms, Genetic variants, SNPs, TNF inhibitor, Biologics, Etanercept, Adalimumab, Infliximab, DMARDs, nbDMARDs, Methotrexate, Sulphasalazine, Leflunomide, Hydroxychloroquine, Ciclosporin, NSAIDs

## Abstract

TNF-blocking agents, non-biological disease-modifying anti-rheumatic drugs (nbDMARDs) and non-steroidal anti-inflammatory drugs (NSAIDs) are commonly prescribed treatments in psoriatic arthritis. A large proportion of patients do not respond to these medications, although unfortunately clinically useful biomarkers that predict future response are currently lacking. Several candidate gene polymorphisms have been associated with responses to biologic therapies and nbDMARDs; however, replication and validation of these variants in large prospective psoriatic arthritis cohorts are required before translating these to clinical practice. In this review, we discuss the advances made in pharmacogenetics of treatment response in psoriatic arthritis to date, with focus on biologic therapies approved for use, nbDMARDs and NSAIDs, as well as outline emerging methodologies to obtain data that will help inform a future precision medicine approach in this condition.

## Introduction

Psoriatic arthritis (PsA) is an inflammatory arthritis, with an estimated overall prevalence of 0.3–1 % [1]. PsA represents a heterogeneous disease with a spectrum of disease severity and expression. This may range from mild symptoms requiring minimal intervention to a rapidly destructive and disabling course leading to loss of function. To preserve long-term quality of life, treatment strategies with the aim of abrogation of inflammation and eventual remission/low disease activity are recommended, with evidence supporting a treat-to-target approach similar to rheumatoid arthritis [2•, 3, 4•]. Although the last decade has seen a number of paradigm changes in the therapeutic options for PsA, response to treatment is highly variable. Current therapeutic strategies include non-steroidal anti-inflammatory drugs (NSAIDs), non-biologic disease-modifying anti-rheumatic drugs (nbDMARDs) and biologic agents such as tumour necrosis factor inhibitors (TNFis). Although TNFi agents are highly effective treatments, they cost £10,000 per patient per year in the UK and up to 30–40 % of patients do not respond [5, 6]. The wide inter-individual variability in drug efficacy and toxicity to all agents has meant that clinicians are reliant on a ‘trial and error’ approach and sequential intervention as per their national consensus recommendations. However, by targeting treatments to the strata of patients most likely to respond (stratified or precision medicine), they could be utilised more cost-effectively, as well as increasing the success rates of first-line treatments and fast-tracking PsA patients destined to require biologic therapies to prevent irreversible joint damage.

Biomarkers in PsA to predict disease prognosis and treatment response are currently lacking in clinical practice. A biomarker is a biological characteristic that is measured and evaluated objectively as an indicator of normal biological processes, pathogenic processes or pharmacological response to therapeutic intervention [7]. The availability of sensitive, specific, reproducible and predictive biomarkers in PsA would have immediate implications for a precision medicine approach as well as better tailoring of emerging new treatments. Most recently, a panel of synovial proteins was found to predict response in a small number of adalimumab-treated PsA patients and in combination acting as potential biomarkers [8]. The transition from conventional approaches to better personalisation utilising biomarkers, in selecting the most efficacious treatment with the least adverse events for a given individual, will likely require consolidated expertise from a number of sectors. One such approach is pharmacogenetics, which can be defined as the study of the variability in drug response because of heredity [9]. Specific advantages of pharmacogenetics are that genetic variants are inexpensive to assay, are stable over time and can provide clues to causality and pathogenesis [10•]. Most importantly, they occur prior to commencing treatment rather than being produced as a consequence [10•], with therefore promising potential for use as predictive biomarkers. Herein, we review the progress that has been made to date in the identification of genetic biomarkers of pharmacological treatment response in PsA, focussing on biologics approved for use, nbDMARDs and NSAIDs, with reference to other chronic inflammatory disease pharmacogenetic studies where appropriate.

## Genetic Predictors of Response to Biologic Therapy

### Tumour Necrosis Factor Inhibitors

Biologic agents are being used increasingly to treat moderate to severe PsA patients in whom conventional treatments have failed. There are two broad classes of TNFis approved for use in PsA: monoclonal antibodies to TNF (infliximab, adalimumab, golimumab, certolizumab pegol) and the TNF fusion protein receptor inhibitor (etanercept). A limited number of pharmacogenetic studies to predict TNFi treatment response have been conducted to date specifically in PsA. All such studies in PsA have used a candidate gene approach, where genes are selected for investigation based on knowledge of the biological pathways they lie on and the therapeutic agent to which response is being assessed.

Polymorphisms within the *TNF* promoter region have been identified to influence clinical efficacy of etanercept in a study that combined patients with rheumatoid arthritis (RA), ankylosing spondylitis and PsA [11] (Table 1). In this study, the −308 G/G (rs1800629) genotype in the promoter of the TNF gene conferred a better response to treatment than A/A or A/G genotypes. Only 10 patients with PsA were included; however, it is promising to note a meta-analysis of similar studies of this polymorphism, which included 692 RA patients treated with infliximab, adalimumab or etanercept, showed that the −308(A) variant predicts poor response to TNFis. In the latter analysis, irrespective of the prescribed TNFi, the frequency of the *TNF* –*308A* variant was 22 % in responders, compared with 37 % in non-responders [OR = 0.4, 95 % confidence interval (CI) 0.4–0.7; *P* = 0.000245]. In another small PsA study (*n* = 58), which assessed single-nucleotide polymorphisms (SNPs) at positions −238 (rs361525), −308 and −489 (rs80267959) of the TNF-α gene in response to TNFis, the −489A allele showed a trend of association with PsA response to etanercept (not significant). However, in that Italian Caucasian population, the association with the −308 G/G genotype was not replicated [12].

**Table 1:** Impact of candidate genes on treatment response in psoriatic arthritis

Therapy	Author	Candidate gene/polymorphism	Sample size (*n*)	Primary outcome	Maximum follow-up (months)	Genotype/phenotype correlation	Protein implicated
Biologic therapies							
Etanercept	Seitz et al. [9]	*TNF*-*α*-*308*	54 (*n* = 10 PsA patients)	DAS28	6	*TNF*-*α*-*308GG* > *A* associated with better response	TNF
Infliximab, adalimumab, etanercept	Murdaca et al. [10•]	*TNF*-*α*-*489A*	58	ACR 20	6	*TNF*-*α*-*489A non-significantly* associated with better response to etanercept only	TNF
Infliximab	Morales-Lara et al. [13]	*TNF R1A*	55	EULAR response	3	*TNFR1 AA* > *AG*/*GG* associated with better response	TNFR1
		*TRAIL*-*R1*	55	EULAR response	6	*TRAILR1 CC* > *CG*/*GG* associated with better 6-month response	TNF-related apoptosis-inducing ligand receptor 1
Infliximab, adalimumab, etanercept	Ramírez et al. [15]	*FCGR2A*	103	EULAR response	6	High-affinity *FCGR2A*-131H allele (HH + HR) associated with better response to etanercept only	FCGR
Infliximab	Morales-Lara et al. [16]	*FCGR3A*	90 (*n* = 16 PsA)	ACR20	3	High-affinity *FCGR3A* V158F (FV + VV > FF) *non-significantly* in PsA patients only	FCGR
Non-biologic DMARDs							
Methotrexate	Chandran et al. [29]	*DHFR*	119	50 % reduction in swollen joint count	6	*DHFR* 35289A associated with better response	Dihydrofolate reductase

*ACR20* American College of Rheumatology 20, *DAS28* disease activity score of 28 joints, *DMARDs* disease-modifying anti-rheumatic drugs, *TNF* tumour necrosis factor

The TNF receptor 1A (TNFR1A) variant rs767455/G36A in PsA patients has been associated with a better European League Against Rheumatism (EULAR) response at 3 months to infliximab both with the AA genotype (AA 85 % vs. AG/GG 58.9 %; *P* = 0.04) and with the A allele (A 76.7 % vs. G 58.3 %; *P* = 0.03) [13]. Interestingly, this was in the opposite direction to RA patients in the same study, whilst a Crohn’s disease cohort also found biological response to infliximab was lower in patients carrying TNFR1 36G mutation in the *TNFR1* gene [14]. In the same study [13], TNF-related apoptosis-inducing ligand receptor 1 (*TRAIL*-*R1*) rs20575 CC genotype was also associated with a 6-month EULAR response to infliximab in PsA (Table 1). In psoriasis, polymorphisms in *TNFAIP3* are associated with response to TNFis, with the SNPs (rs2230926 and rs610604) acting as markers of beneficial response to three TNFis tested [15]. *TNFAIP3*, which has also been identified as a susceptibility locus for PsA [16], is a gene encoding the A20 protein, a TNF-α-inducible zinc finger protein thought to limit NF-κB-mediated immune responses. Therefore, there is some evidence that polymorphisms affecting TNF may affect treatment response to TNFis in PsA patients; however, this may vary according to disease and drug subtype, and larger validation studies are required to confirm these associations.

The presence of the high-affinity *FCGR2A*-*131H* allele in either homo/heterozygous combinations (HH and HR) in PsA patients receiving etanercept showed a strong trend to a higher rate of EULAR response compared with those without a response (93 vs. 67 %; *P* = 0.034) [17]. *FCGR2A*, located on chromosome 1, encodes one member of a family of immunoglobulin Fc receptor genes found on the surface of many immune response cells. The protein encoded by this gene is a cell surface receptor and is involved in the process of phagocytosis and clearing of immune complexes. A smaller PsA study, which evaluated the specific distribution of the *FCGR3A V158F* polymorphism in relation to infliximab response at 3, 6, and 12 months, found that more patients with a high-affinity genotype (FV + VV) achieved a EULAR response at 3 months (20 % FF vs. 83.3 % FV-VV; *P* = 0.036, *P* = 0.067) [18]. This association however was not significant, limited by the small numbers of patients in the study and hence study power.

The prevalence of axial involvement amongst patients with PsA has been reported to be between 25 and 70 %, depending on the criteria used for its definition [19], and PsA is also known to share susceptibility loci with ankylosing spondylitis (AS) [20, 21•]. Few studies have analysed the role of genetic markers in the response to TNFi therapy in AS patients, but the majority have shown an association of favourable response with HLA-B27 status [22, 23]. Outside the MHC, one study suggested variants within macrophage migration inhibitory factor (*MIF*) gene (rs755622), interleukin 18 receptor accessory protein (*IL18RAP*) gene (rs917997), *IL10* (rs1800896), TNF receptor superfamily member 1B [(*TNFRSFB1*); rs4355801] and ADP-ribosylation factor GTPase-activating protein 2 (*ARFGAP2*) gene (rs3740691), were associated with non-response in 121 AS patients [24]. These results are yet to be replicated independently.

In RA, only two associations with TNFi treatment response have approached genome-wide significance (*P* < 5 × 10^−8^) and/or have been replicated in independent cohorts. These include a polymorphism in the *CD84* gene (rs6427528; G > A) which encodes SLAM family member 5 and has been associated with reduced response to etanercept [25]; the *PDE3A*–*SLCO1C1* locus (rs3794271; C > T) was associated with reduced efficacy to the TNFis etanercept, infliximab and adalimumab [26]. Larger homogenous cohorts are required to more accurately assess and replicate these variants to evaluate if they can predict response to TNFis specifically in PsA patients, to inform future clinical decisions regarding treatment selection.

## Genetic Predictors of Response to Other Treatment

### Non-biologic Disease-Modifying Anti-rheumatic Drugs

#### Methotrexate

Methotrexate, a folate antagonist, is the most commonly used systemic nbDMARD in PsA. Although the mechanism of action in PsA is not fully understood, methotrexate requires intracellular uptake and inhibits enzymes of the folate, purine and pyrimidine pathways. Given the considerable inter-individual variability in response (and approximately 30 % of treated individuals developing hepatotoxicity [27] or gastrointestinal adverse events), reliable biomarkers to predict response at the outset would be extremely beneficial to help optimise current treatment regimens.

Pharmacogenetic studies assessing methotrexate response specifically in PsA are sparse. The gene polymorphisms which influence metabolism of methotrexate may be classified into those that influence methotrexate transport across the cell membrane and those that influence enzymes in the cellular pathway of methotrexate [28]. One study, which assessed 119 PsA patients, evaluated associations between effectiveness, toxicity, and drug survival and polymorphisms of genes coding for the folate pathway enzymes methylenetetrahydrofolate reductase (MTHFR), dihydrofolate reductase (DHFR) and reduced folate carrier (RFC) [29]. A polymorphism in the *DHFR* gene was associated with better methotrexate response (Table 1). The DHFR enzyme converts dihydrofolate to tetrahydrofolate, required for DNA synthesis and cell growth. Although DHFR is inhibited by methotrexate, it is unclear if this inhibition is crucial to its anti-inflammatory effects. Interestingly, PsA patients homozygous for the minor allele of *MTHFR* 677C/T (677TT (rs1801133)) had more liver toxicity [29]. Studies in RA investigating polymorphisms in the *MTHFR* gene, as predictors of response to methotrexate, have reported conflicting results [30, 31]. A previous meta-analysis evaluating key polymorphisms of C677T (rs180113) and A1298C (rs1801131) within *MTHFR* established they were not reliable predictors of treatment response, although it was acknowledged that there was substantial heterogeneity within the studies [32].

#### Sulphasalazine

Sulphasalazine can be effective for joint pain and skin disease in PsA [33, 34]. Following ingestion, a small amount is absorbed systemically, whilst the majority is reduced by intestinal bacteria to 5-aminosalicylic acid and sulphapyridine. In the liver, an acetate group is added to the sulphapyridine by the phase II enzyme *N*-acetyltransferase 2 (NAT2) to form *N*-acetyl sulphapyridine, which subsequently undergoes renal excretion [35]. NAT2 is encoded by an 870-bp gene (*NAT2*), and 40–70 % of individuals are either homozygous or compound heterozygous for *NAT2* polymorphisms [36], which influences whether individuals are rapid, intermediate or slow acetylators. Most NAT2 variant alleles include one or more of the following seven most frequent SNPs: rs1801279 (191G > A), rs1041983 (282C > T), rs1801280 (341 T > C), rs1799929 (481C > T), rs1799930 (590G > A), rs1208 (803A > G) and rs1799931 (857G > A) [37]. With rapid acetylators having lower plasma concentrations, they may be less prone to adverse events, however may require higher doses to maintain efficacy. The main route of elimination of sulphasalazine is faecally, which is mediated by ATP-binding cassette protein G2 (ABCG2), a transmembrane protein that transports sulphasalazine into the intestinal lumen [38].

A recent study that evaluated 229 patients with early RA on sulphasalazine (+/− combination therapy) revealed that *NAT2* acetylator status (rs1041983) was associated with higher incidence of adverse events but not associated with efficacy [38]. Interestingly, the *ABCG2* genotype (rs2231142) was associated with remission in RA (odds ratio 3.34 (95 % CI 1.18–9.50), *P* = 0.024), even after adjustment for confounders and in the sensitivity analysis which assessed Caucasian patients only. A small study also suggested genetic polymorphisms of *NAT2* leading to differences in acetylator status may account for variation in the response to sulphasalazine in patients with discoid lupus [39]. Furthermore, studies in RA [40, 41] and most recently in ankylosing spondylitis [42] suggest *NAT2* variants, which lead to slow acetylator status, may contribute to adverse events in sulphasalazine-treated patients. These results however do require replication in independent cohorts, and further work needs to be performed to extrapolate these results to PsA patients.

#### Leflunomide

Leflunomide inhibits de novo pyrimidine synthesis, leading to inhibition of T cell proliferation and activation, potentially targeting the underlying pathophysiologic events in PsA, psoriasis and RA. Its metabolite teriflunomide causes non-competitive and reversible inhibition of dihydroorotate dehydrogenase (DHODH), the key enzyme required for pyrimidine synthesis [43]. In RA, a number of studies have evaluated polymorphisms within the *DHODH* gene: Firstly, Pawlik et al. studied 147 RA patients on leflunomide monotherapy and found that remission was more frequent in patients who carried a common missense polymorphism in the coding region (rs3213422 19 C > A). A small study assessing disease activity in 56 RA patients at 3 months suggested carriage of a six-marker *DHODH* haplotype was associated with reduced treatment response in the short term [44]. Finally, a retrospective RA study in 105 patients suggested *DHODH A40C* was associated with a 6.8-fold increased risk of overall toxicity related to the drug; however, efficacy was not assessed [45].

In vitro studies have suggested oestrogen interferes with suppression of cytokine production by leflunomide [46, 47]. Oestrogen acts through the receptors ESR1 and ESR2, and the genes encoding for them are responsible for transducing extracellular signals into transcriptional responses. Polymorphisms within the genes encoding the oestrogen receptors (*ESR1* and *ESR2*) in women have been therefore investigated. In a study carried out on 115 women on leflunomide, a better response to treatment in patients was observed with *ESR1* rs9340799 AA and rs2234693 TT genotypes after 12 months of therapy (a worse response with the G-C haplotype) [48]. Teriflunomide also inhibits tyrosine kinases, including p56LCK and p59FYN, both of which are candidate substrates for the PTPN22–CSK complex. More recently, the *PTPN22* genotype (rs2476601), known to be a strong susceptibility locus for RA, was evaluated as a predictor of response/toxicity to leflunomide therapy in a small group of RA patients in which no association was found [49].

A previous study on human liver microsomes suggested that cytochromes (CYP450) are involved in leflunomide activation, and the major enzymes responsible for leflunomide metabolism are CYP1A2, CYP2C19 and CYP3A4 [50]. In a prospective RA study, which compared 62 patients who tolerated the drug vs. 43 who discontinued due to adverse events (6 due to inefficacy), the CYP1A2*1F (*CC* > *A*) had a 9.7-fold higher risk for overall leflunomide-induced toxicity [51]. In a retrospective cohort study of 78 patients, the *CYP2C19* phenotype was associated with cessation due to toxicity [52], most of whom were also on a concomitant DMARD, although these results conflict with the findings of previous studies [45, 53]. To date, there have been no studies assessing the pharmacogenetics of leflunomide in PsA. Current evidence suggests a possible diagnostic value of genotyping patients with RA as biomarkers, although future studies need much larger sample sizes, longer follow-up and replication of the above variants and across disease areas such as PsA.

#### Other Non-biologic Disease-Modifying Anti-rheumatic Drugs

Other nbDMARDs infrequently used in the treatment of PsA include hydroxychloroquine and ciclosporin. There are no pharmacogenetic studies evaluating hydroxychloroquine as monotherapy in inflammatory arthritis. Ciclosporin polymorphisms have been evaluated previously in paediatric transplantation candidates only [54] and most recently to assess 3-month treatment response of psoriasis patients [55]. In the latter study, the *ABCB1* C3435T (rs1045642) polymorphism was found to be associated with a reduced response to ciclosporin, in both SNP and haplotype analyses. ABCB1 is known to affect P-glycoprotein levels, lower levels of which may impair drug transport and thus reduce efficiency of therapy.

### Non-steroidal Anti-inflammatory Drugs

NSAIDs are frequently used as adjunct treatments in PsA for pain relief and improving inflammatory symptoms. A subgroup of PsA of patients will experience worsening of their psoriasis when on this class of drugs. The metabolism of NSAIDs is highly regulated by the cytochrome P450 enzymes, which comprise a category of proteins whose biosynthesis is controlled by a large superfamily of genes. NSAIDs are primarily metabolised by the polymorphic *CYP2D9* gene, which has over 70 variable alleles and may vary from each other in only one nucleotide [56]. *CYP2D9* allele frequency is variable across ethnicities with the functional group of alleles predominant in approximately 70 % of European Caucasians, whilst Asians and Pacific Islanders have a high frequency (∼40 %) of a reduced function allele, CYP2D6*10, leading to slower drug metabolism [57]. Individuals with several variant alleles (such as CYP2C9*2 and CYP2C9*3) have demonstrated decreased metabolic clearance compared with individuals with the wild-type enzyme (CYP2C9*1) [58]; however, prediction of NSAID drug dosing regimens based on genotyping alone remains challenging. To date, there are no specific studies of *CYP2C9* genotypes as predictors of PsA response or to identify in which individuals psoriasis may worsen.

## Challenges and Future Directions

With emerging novel biologic targets and expansion of the PsA therapeutic armamentarium, the identification of reliable and predictive genetic markers of therapeutic response to optimise the cost/benefit ratio of these expensive yet effective drugs has become increasingly important. Unfortunately, large-scale studies performed in ethnically homogenous populations and in PsA patients followed up for an adequate period of time are lacking; therefore, pharmacogenetic studies have been underpowered to translate these findings to clinical practice. Several issues confound interpretation of the outlined studies. PsA is a complex polygenic disease, with significant phenotypic heterogeneity. Outcome measures used to measure treatment response can be highly inconsistent between studies, making results in the same disease subset difficult to extrapolate. An effort to standardise such measures is currently underway by the Outcome Measures in Rheumatoid Arthritis Clinical Trials (OMERACT) PsA special interest group [59], which will be helpful to inform future core minimal set of outcomes not only in trials but also in longitudinal studies. In some cases, genetic polymorphisms identified as potential predictors of treatment response may also be markers of more severe disease, leading to an increased response to TNFi agents (normally prescribed to the most severely affected PsA individuals).

The multifactorial nature of treatment response make small genetic effects difficult to discover even from genome-wide association studies, which have not yet been conducted for this purpose in PsA. The search for genetic variations influencing therapeutic response may also be affected by factors not related primarily to a drug’s mechanism of action. An important mechanism for treatment failure of biologics is immunogenicity or the formation of anti-drug antibodies, particularly in response to monoclonal antibodies. In the case of adalimumab, they may be formed in around 22 % of PsA patients and lead to treatment failure [60]; their occurrence is further influenced by use/dose of concomitant nbDMARDs such as methotrexate amongst other factors [61]. Etanercept does not appear to develop detectable anti-drug antibodies that neutralise the effect of the drug; therefore, differential predictive genetic variants in patients treated with this drug alone may be plausible. Furthermore, immunogenicity in PsA may itself vary between carriers of undiscovered genetic factors, and these influences could contribute to genetically based loss of response. Additional important issues in determining treatment response include adherence to rheumatic drugs, which may be as low as 30 % [62, 63], and a current lack of standardised methods of measurement to facilitate comparison between studies.

In the future, a precision medicine approach in PsA prescribing is likely to require integration of serological markers, pharmacological biomarkers as well as emerging technologies and methodologies currently being used in other disease areas. Epigenetic changes are inheritable modifications to DNA not caused by variation in its primary nucleotide sequence and may lead to DNA methylation changes and histone modification affecting treatment response as seen in RA [10•]. A complementary approach in the identification of predictive markers is whole-genome expression profiling or transcriptomics which provides information on which genes are actively being expressed at a given time. In biologic-treated RA patients, the interferon signature has been correlated with clinical response (reviewed previously by Smith et al. [64]). Although epigenetics and transcriptomics in determining PsA treatment response remain unexplored, future work investigating these aspects offers exciting potential for correlating genetics and gene expression data with treatment outcomes.

Expensive drugs such as biologics with high rates of primary/secondary non-response and medications with a narrow therapeutic index leading to severe adverse effects (e.g. certain nbDMARDs) are to be the ideal candidates for exploration of pharmacogenetic biomarkers. Psoriasis and PsA susceptibility loci [20, 21•] could be targeted in future candidate gene studies, given some evidence that RA susceptibility genes associate with response [65]. In the future, the development of a ‘pharmacogenetic signature’ [20] which incorporates multiple genetic variants that predict the efficacy and toxicity of a given drug, combined with emerging biomarkers as above, could contribute to a personalised medicine approach in PsA and tailoring of these expensive drugs to an individual patient.

## Conclusion

In summary, although pharmacogenetics in PsA is still in its infancy, it holds promise towards introducing a precision medicine approach in clinical practice. Given the advances in genetic technology and new targeted anti-cytokine therapies emerging, there will be real potential for translating genetic biomarkers from bench to bedside. However, future studies will need to be adequately powered, and results replicated and validated in independent cohorts, with adequate health economic evaluation before they can be adopted widely in clinical practice. The need to treat inflammatory arthritides such as PsA with the right treatment first time to prevent long-term joint damage and disability, the variable response and toxicity to treatments, the current cost of effective treatments such as TNFis and the emergence of new biologics for its treatment make PsA a worthwhile disease model to assess the utility of a precision medicine approach. In the future, it will be imperative to encompass known demographic, serological, immunological and genomic biomarkers and correlate with robust outcome measures when assessing the additional benefits of prospective genetic testing.
